# *In Vivo* Evaluation of the Antioxidant Activity and Protective Action of the Seaweed *Gracilaria birdiae*

**DOI:** 10.1155/2018/9354296

**Published:** 2018-08-01

**Authors:** Joanna Angelis Costa Barros-Gomes, Daiany Laise Araújo Nascimento, Ana Cristina Rodrigues Silveira, Rayanne Kelly Silva, Dayane Lopes Gomes, Karoline Rachel Teodosio Melo, Jailma Almeida-Lima, Rafael Barros Gomes Camara, Naisandra Bezerra Silva, Hugo Alexandre Oliveira Rocha

**Affiliations:** ^1^Laboratório de Biotecnologia de Polímeros Naturais (Biopol), Centro de Biociências, Departamento de Bioquímica, Universidade Federal do Rio Grande do Norte (UFRN), Av. Sen. Salgado Filho 3000, 59072970 Natal, RN, Brazil; ^2^Departamento de Nutrição, Centro Universitário do Rio Grande do Norte (UNI-RN), Rua Prefeita Eliane Barros, 2000 Tirol, 59014-545 Natal, RN, Brazil; ^3^Instituto Federal de Educação, Ciência e Tecnologia do Piauí (IFPI), São Raimundo Nonato, 64.770-000 Piauí, PI, Brazil; ^4^Escola Multicampi de Ciências Médicas, Universidade Federal do Rio Grande do Norte (UFRN), Av. Cel. Martiniano 541, 59300-00 Caicó, RN, Brazil; ^5^Laboratório de Histologia, Centro de Biociências, Departamento de Morfologia, Universidade Federal do Rio Grande do Norte (UFRN), Av. Sen. Salgado Filho 3000, 59072970 Natal, RN, Brazil

## Abstract

The red seaweed *Gracilaria birdiae* (GB) is farmed and used as food in northeast Brazil. However, the economic potential of this seaweed has been explored little. To enable direct consumption and/or product diversification from GB, it is necessary to evaluate its effect *in vivo*. In this study, the food of mice was improved with the addition of *GB*. After 21 days, the consumption of seaweed reduced the weight gain and blood glucose levels in mice. In addition, it increased the trolox equivalent antioxidant capacity and glutathione reductase and catalase levels compared to those of the control group. In addition, some mice also received carbon tetrachloride (CCl_4_). In this case, histological, enzymatic, and antioxidant tests showed that the seaweed could protect animals from damage caused by this toxic agent. In addition, GB aqueous extract (AE) inhibited 50% of 3T3-L1 cell differentiation into adipocytes, whereas GB ethanolic extract was not effective. AE is composed mainly of sulfated polysaccharides. The results of the present study indicate that the alga GB protected the mice from CCl_4_-induced damage, indicating that the seaweed exhibits protective action *in vivo*. In addition, GB decreased the animal weight gain, which was mainly due to the action of the sulfated polysaccharides synthesized by this seaweed.

## 1. Introduction

Antioxidants exert positive effects on human health, as they protect the human body against harmful effects caused by reactive oxygen species, which damage macromolecules such as membrane lipids, proteins, and DNA and can lead to the development of several diseases, such as cancer and neurodegenerative, inflammatory, and heart diseases [1, 2].

Recently, interest in the development of antioxidants from natural sources of marine flora and fauna [3] has increased considerably in the food and pharmaceutical industries. Red seaweeds are one of the richest sources of natural antioxidants, including phenolic compounds, vitamins, and sulfated polysaccharides [4, 5]. In addition, in red seaweeds, insoluble fibers are composed of cellulose and the soluble fibers are composed of sulfated galactans or soluble xylans. Fibers are mainly used as bulking and texturing agents, which are essential for the development of low-calorie foods. A high intake of dietary fiber reduces the development of chronic diseases, such as diabetes, obesity, heart diseases, and cancer [6].

Cappo and colleagues [7] described the genus *Gracilaria* (red seaweed) as the most promising marine source of polysaccharides because of its ability to produce sulfated galactans in large quantities. This genus is distributed across the globe, in tropical and subtropical climate zones. To date, more than 300 species of *Gracilaria* have been identified, of which 160 species have been taxonomically accepted [8]. Some species have been grown commercially in countries that cultivate such seaweeds, including edible *Gracilaria edulis* in India [8].

*Gracilaria birdiae* is a common seaweed of the northeastern coast of Brazil. Infrared and nuclear magnetic resonance have been used to elucidate the major structural features of the sulfated galactan synthesized by *G. birdiae* [9]. This galactan is mainly composed of alternating residues of 3-linked-*β*-D-galactopyranose (G unit) and 4-linked-3,6-anhydro-*α*-L-galactopyranose (LA unit). The G unit is mainly substituted with either a methyl or a sulfate ester group [9]. Subsequently, the anti-inflammatory effects of this sulfated galactan have been demonstrated *in vivo* [10], and this polysaccharide prevents naproxen-induced gastrointestinal damage in rats [11]. It also exhibits antioxidant activity [12]. However, the economic potential of this seaweed is little explored.

In addition to being a source of agar, *G. birdiae* is also used by some poor populations as a source of animal and human food. Therefore, it is cultivated in several places [13]. To enable direct consumption and/or product diversification from *G. birdiae*, it is necessary to evaluate its effect *in vivo*. In this study, mouse food was supplemented with *G. birdiae* or vitamin E and after the treatment period, antioxidant parameters, including antioxidant enzymatic activity, were evaluated. In addition, some groups were supplemented with carbon tetrachloride (CCl_4_) in order to evaluate the capacity of the seaweed to protect animals from damage caused by this toxic agent.

## 2. Materials and Methods

### 2.1. Materials

Acetic acid, Folin-Ciocalteu phenol reagent, ethanol, and sulfuric acid were obtained from Merck (Darmstadt, Germany). Monosaccharides, 3-isobutyl-1-methylxanthine (IBMX), insulin, dexamethasone, and bovine serum albumin (BSA) were purchased from Sigma-Aldrich Co. (St. Louis, MO, USA). Sodium bicarbonate, culture medium components (minimum essential Dulbecco's modified Eagle's medium (DMEM)), nonessential amino acids, fetal bovine serum, sodium pyruvate, and phosphate-buffered saline (PBS) were purchased from Invitrogen Canada Inc. (Burlington, ON, Canada). Water was purified with a Milli-Q system (Millipore®, Bedford, MA, USA). All other solvents and chemicals were of analytical grade.

### 2.2. Raw Material

The red seaweed, *G. birdiae*, was collected in Rio do Fogo Beach (Rio Grande do Norte, Brazil, 5°16′16.61^″^S/35°22′54.29^″^W) by fishermen from the community. The seaweed was sun-dried and then taken to the laboratory for cleaning to eliminate residue and epiphytes. The material was powdered, and then, 200 mL of ethanol was applied five times overnight to reduce pigments, according to the protocol described by Fidelis and colleagues [12]. The supernatant was removed, and the red seaweed was dried at 50°C under ventilation. The dried material was packed in polyethylene bags and stored at room temperature (28°C) in the dark.

### 2.3. Extracts

The ethanolic extract (EE) was obtained as follows: 5 g of dried material (seaweed) was suspended in 50 mL of 80% ethanol by shaking for 24 h in the dark. The solution was filtered and evaporated to dryness under reduced pressure. The dried powder was dissolved in 50% distilled water and ethanol (*v*/*v*) and stored at −20°C until use. The extraction yield was 0.1%.

The aqueous extract (AE) was obtained as described by Fidelis and colleagues [12]. Briefly, 5 g of seaweed was suspended in 0.25 M NaCl (50 mL), and the pH was adjusted to 8.0 with NaOH. Next, 900 mg of Prolav 750 (Prozyn Biosolutions, São Paulo, SP, Brazil), a mixture of alkaline proteases, was added for proteolytic digestion. After incubation for 24 h at 60°C under agitation and periodical pH adjustments, the mixture was filtered through cheesecloth. The filtrate was also collected by centrifugation (10,000 ×g, 20 min), vacuum-dried, resuspended in distilled water, and stored at −20°C until use. The extraction yield was 1.5%.

### 2.4. *In Vivo* Antioxidant Tests

Thirty-six male mice *(Mus musculus)* weighing 30–35 g, aged 30 days, purchased from Biotério de Reprodução Animal do Centro, Universitário do Rio Grande do Norte, were used in this study. After the acclimation period, the mice were weighed and randomly divided into six groups, each containing six mice. The control group received a saline solution (0.9%, *w*/*v*) by gavage. Mice in the second group received seaweed (6 mg/kg body weight) orally in 200 *μ*L of saline solution, daily. The third group received vitamin E (50 mg/kg body weight) daily. Animals of the fourth group received CCl_4_ : mineral oil (1 : 1.2 mL/kg body weight/day). The fifth group received a daily oral dose of seaweed (6 mg/kg body weight), 200 *μ*L in saline solution, followed by CCl_4_ : mineral oil (1 : 1.2 mL/kg body weight/day) 30 minutes later. Mice of the sixth group received a daily oral dose of vitamin E (50 mg/kg body weight) followed by CCl_4_ : mineral oil (1 : 1.2 mL/kg body weight/day) 30 minutes later. Animals in the fourth, fifth, and sixth groups received CCI_4_ every other day. After 21 days of treatment, the mice were weighed and their blood glucose levels were measured using tail blood and a glucometer (On Call® Plus: San Diego, California, USA). Finally, mice were euthanized as described above.

After euthanasia, the kidneys and livers of the animals were washed with PBS, pH 7.4, to remove any red blood cells and clots. Then, samples were divided into two equal parts. One part was used for histological analysis, and the other was used in the following protocol: the tissues were homogenized on ice in 5–10 mL of cold buffer (i.e., 50 mM potassium phosphate, pH 7.0, containing 1 mM EDTA) per gram of tissue and centrifuged at 10,000 ×g for 15 min at 4°C. Then, the supernatants were removed, frozen on dry ice, and stored at −80°C until analysis.

The biological assay was developed in accordance with the ethical principles in animal experimentation, and the project was approved by the Ethics Committee on Animal Use (UFRN—Protocol 059/14).

#### 2.4.1. Evaluation of Kidney and Liver Homogenate Antioxidant Capacity

To determine antioxidant capacity, the trolox equivalent antioxidant capacity (TEAC) assay was used, as described by Seeram et al. [14]. For this, we used 7 mM 2,20-azino-bis(3-ethylbenzothiazoline-6-sulfonate) (ABTS; 5 mL) and 140 mM potassium persulfate (0.088 mL). After 16 h of darkness (25°C), the solution was diluted with ethanol (98%) until the absorbance at 734 nm was 0.7 ± 0.05. Then, 2 mL of the diluted ABTS solution was added to the samples (0.02 mL). The absorbance was measured after 6 min at 734 nm. TEAC was expressed in trolox equivalent per 100 *μ*L of tissue extract.

#### 2.4.2. Superoxide Dismutase Activity

Superoxide dismutase (SOD) activity was analyzed by spectrophotometry, as described previously [15]. SOD activity is expressed as the amount of enzyme required to inhibit the rate of nitroblue tetrazolium (NBT) oxidation by 50%.

#### 2.4.3. Catalase Activity

Catalase (CAT) activity was determined as described by Aebi [16]. Reaction with hydrogen peroxide was observed using a spectrophotometer (240 nm, 1 min, 25°C). Enzymatic activity was determined as the unit of activity corresponding to the nmol of destroyed H_2_O_2_/min/mL.

#### 2.4.4. Glutathione Reductase Activity

Glutathione reductase (GR) activity was assessed as described by Carlberg and Mannervik [17]. The reaction with TNB was observed by using a spectrophotometer (412 nm). GR activity was determined as the unit of activity corresponding to nmol/min/mL.

### 2.5. Necropsy and Histological Analysis

Animals were euthanized by administration of high doses of anesthetic (20 mg/kg thiopental). The tissues and organs were examined macroscopically for visible abnormalities. Subsequently, the liver and kidneys of all animals were removed, weighed, and divided into two equal parts. For histological analyses, one of these parts was fixed in buffered formaldehyde. After 24 h, the apparatus was embedded in paraffin, sectioned (5 *μ*m in diameter), placed on glass slides, and stained with hematoxylin and eosin. The slides were examined through optical microscopy (20, 40, and 100x objective lens).

### 2.6. Chemical Analysis and Monosaccharide Composition

Sulfate content was determined according to the gelatin-barium method as previously described [18], using sodium sulfate (1 mg/mL) as a standard and following acid hydrolysis of the polysaccharides (4 M HCl, 100°C, 6 h). Protein content was measured using the Spector's method [19]. The polysaccharides were hydrolyzed with 0.5, 1, 2, and 4 M, respectively, for various lengths of time, (0.5, 1, 2, and 4 h), at 100°C. Reducing sugars were determined using the Somogyi-Nelson method [20]. After acid hydrolysis, sugar composition was determined by a LaChrom Elite® HPLC system from VWR-Hitachi with a refractive index detector (RI detector model L-2490). A LichroCART® 250-4 column (250 × 40 mm) packed with LiChrospher® 100 NH_2_ (5 *μ*m) was coupled to the system. Overall, 0.2 mg of sample was used, and the analysis proceeded for 25 min. The following sugars were analyzed as references: arabinose, fructose, fucose, galactose, glucose, glucosamine, glucuronic acid, mannose, and xylose.

Phenolic compounds were quantified by the colorimetric method with the Folin-Ciocalteu reagent using gallic acid as a standard [21].

### 2.7. MTT Assay

The cytotoxicity of extracts was determined using the MTT assay as previously described by do Nascimento et al. [22]. For the tests, 0.5 × 10^4^ cells were grown in 96-well plates with DMEM containing the samples at concentrations of 0.2, and 1 mg/mL for 24, 48, and 72 h (each concentration in triplicate). The cell capacity to reduce MTT was determined by the colorimetric test with 3-(4,5-dimethylthiazol-2-yl)-2,5-diphenyl-tetrazolium bromide (MTT), as described above.

### 2.8. Cell Culture and Differentiation

3T3-L1 preadipocyte cells were purchased from a cell bank in Rio de Janeiro, RJ, Brazil (CR089-BCRJ/UFRJ) and maintained with 10% FBS/DMEM containing 4.5 g/L glucose, 100 U/mL penicillin, 0.1 mg/mL streptomycin, and 0.25 mg/mL amphotericin B at 37°C in a 5% CO_2_ incubator. Confluent cells were differentiated by incubation with the hormone mixture MDI containing 10 *μ*g/mL insulin, 1 *μ*M dexamethasone, and 0.5 mM IBMX, in 10% FBS/DMEM for 72 h. Thereafter, the cells were maintained in postdifferentiation medium containing 10 g/mL insulin in 10% FBS/DMEM, in the presence of samples (0.2 mg/mL), and the medium was replaced every 3 days. The same concentration of extract was supplemented at 3-day intervals when the culture medium was replaced. Differentiation was completed at day 15 as measured using the dye Oil Red O.

### 2.9. Oil Red O Staining and Free Glycerol

Fifteen days after differentiation was induced, cells were stained with Oil Red O. Cells were washed twice with PBS and fixed with 3.7% formaldehyde for 10 min. Fixed cells were stained with 0.2% Oil Red O-isopropanol for 1 h, and the excess stain was washed with 70% ethanol and water. Cells were then photographed using a microscope (Nikon Eclipse Ti-E). Stained oil droplets were dissolved with isopropanol and quantified by spectrophotometric analysis at 510 nm. The optical density of MDI-treated cells was taken as 100% of the relative lipid content. The results were representative as the relative lipid contents of each experimental group.

To determine free glycerol, we used a Sigma kit (Sigma-Aldrich Co., St. Louis, MO, USA). This kit contains adenosine triphosphate, glycerol kinase, glycerol phosphate oxidase, dye reagent, and buffer. The nature of these last two compounds is not reported by Sigma. These components were mixed to obtain the free glycerol reagent that was prepared according to the manufacturer's instructions at room temperature (22°C). For the test, the following were prepared: a solution without samples (10 *μ*L water + 400 *μ*L free glycerol reagent), the standard (0, 2.5, 5, 7.5, and 10 *μ*L glycerol + 400 *μ*L free glycerol reagent, corresponding to 0 mM, 0.25 mM, 0.5 mM, 0.75 mM, and 1.0 mM, resp.), and samples (10 *μ*L sample + 400 *μ*L free glycerol reagent). To complete mixing, the components were shaken by inversion and heated in a water bath at 37°C for 5 min. The absorbance of the mixture was measured at 540 nm for the quantitative enzymatic determination of glycerol. The blank was used as negative control, and the standard was used as a positive control. The procedure involves the enzymatic hydrolysis of triglycerides to glycerol and free fatty acids with lipase. The increase in absorbance at 540 nm is directly proportional to the free glycerol concentration of the sample.

### 2.10. Agarose Electrophoresis

This method involves the separation of molecules according to their interaction with 0.05 M PDA (diamine-propane acetate) buffer and the exposure of negative charges. Agarose gel (0.6%) was prepared with 0.05 M PDA buffer and molded in a blade glass (7.5 cm × 7.5 cm × 15 mm or 5.0 cm × 7.5 cm × 15 mm). Five microliters of each sample containing the extracts was applied to spots present in the gel and subjected to electrophoresis at 90 V in a refrigerated system (4°C). Following electrophoresis, the agarose blade was soaked in 0.1% CTV (cetavlon) for 2 h at room temperature. After 2 h, the blade was exposed to hot air and dried. As soon as the blade was dry, toluidine blue reagent was applied and was stirred every 15 min. Finally, excess dye was eliminated with acetic acid/ethanol/water, and the blade was analyzed [12].

### 2.11. Statistical Analysis

Numerical results are expressed as the arithmetic mean (±standard error). The statistical analyses compared the control group with the test groups for each treatment by using two-way ANOVA followed by the Student-Newman-Keuls test, except in Table 1, when the data were analyzed by two-way ANOVA followed by the Bonferroni method. Cell experiments were tested in triplicate, and the experiment was repeated at least three times. In this case, the statistical analysis was performed by using one-way ANOVA followed by the Turkey-Kramer test. All tests were conducted using SigmaPlot® (Systat Software, San Jose, CA, USA).

## 3. Results and Discussion

### 3.1. Body and Organ Weights

**Figure 1:**
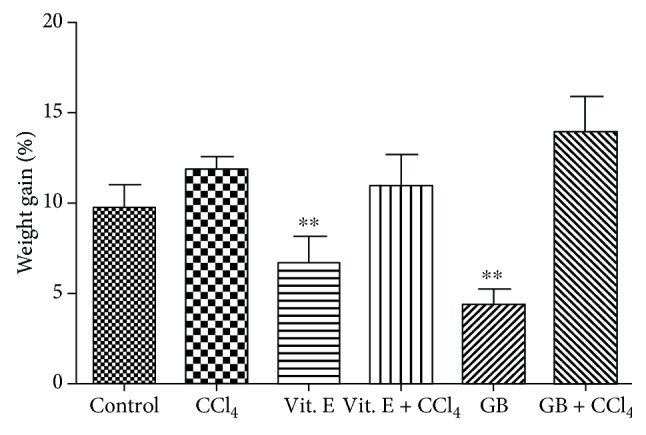
Percentage weight gain of mice after 21 days. Values are expressed as mean ± standard deviation (^∗∗^*p* < 0.01).

The animals were treated for 21 days, after which they were weighed; the obtained data are shown in Figure 1. The weight of animals in the control group (animals with normal diet) increased by approximately 10% compared to that on the first day of treatment (data not shown). There was no statistical difference in the weight gain of the CCl_4_-treated animals (*p* > 0.05) compared to that of the control group animals, as shown in Figure 1. To our knowledge, this is the first study to evaluate the effect of CCl_4_ on animal weight gain.

Animals treated with vitamin E had a significantly smaller gain in weight than control animals. Tocotrienols (T3s), a subclass of vitamin E, were found to promote a reduction in fat mass, body weight, plasma concentrations of free fatty acids, triglycerides, and cholesterol by their action on adipose tissue, modulating energy use in cells of that tissue, adipogenesis, differentiation, apoptosis in preadipocytes, and inflammation [23]. Thus, vitamin E may have a wide range of actions and may act as an antiobesity agent. Although the antiobesity action of vitamin E remains to be clarified, many researchers have stated that this mechanism is directly related to the antioxidant capacity of vitamin E, which would make it a good free radical scavenger and inhibitor of lipid peroxidation. These factors are related to several events that promote weight gain [24, 25]. This hypothesis is consistent with our findings.

In this study, the animals that consumed the GB seaweed had a significantly lower body weight gain than those in the other groups, including the group treated with vitamin E. This suggests that the GB alga may have an antiobesity effect. Several substances with antioxidant action have been suggested as antiobesity agents [23, 26]. In addition, GB exerts antioxidant effects because it can synthesize molecules with antioxidant activity, such as sulfated polysaccharides [9, 12] and phenolic compounds [12]. Thus, we propose that the antiobesity effects of GB, as well as that of vitamin E, are related to their antioxidant capacity.

In addition, animals treated with GB or vitamin E exhibited a significant reduction in glycemia compared with the control group animals (Table 1). This may also be involved in the antioxidant activity of GB and vitamin E.

Notably, GB is rich in fibers, which may be associated with this activity [27]. The American Dietetic Association [28] states that fibers cause slower digestion and intestinal absorption, which increases satiety and consequently reduces food intake and weight gain. The antiobesity effect of other seaweeds is attributed to the presence of fibers in their composition, as reported by Kang and colleagues [26], who verified that rats fed with a lipid-rich diet and the red seaweed *Gelidium amansii* presented lower weight gain than those fed with a hyperlipidic diet.

Therefore, it is possible that the antioxidant molecules of GB and its fibers act together to reduce blood glucose and weight.

In short, our data show that the consumption of GB decreases weight gain and glycemia in animals, and this corroborates the findings of Iwai [29], who stated that seaweed presents antidiabetic effects *in vivo* and may have beneficial properties on the prevention of diabetes.

In this study, GB and vitamin E were not effective at reducing weight gain in the presence of CCl_4_. It is possible that CCl_4_ provides a more intense oxidative environment, which prevents the action of both seaweed and vitamin E.

### 3.2. Evaluation of Antioxidant Capacity *In Vivo*

To determine the antioxidant capacity of GB *in vivo*, the kidneys and liver were removed from mice treated with seaweed; homogenates of these organs were used to perform the TEAC test.

**Figure 2:**
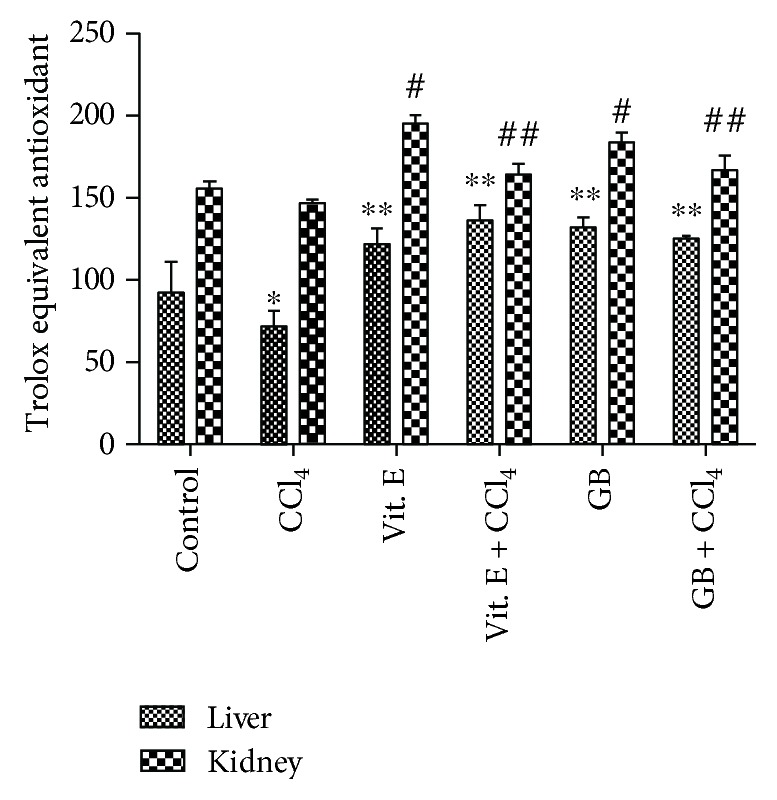
Trolox equivalent antioxidant capacity (TEAC) of kidney or liver homogenates. ∗ and # indicate a significant difference between groups versus control. ^∗^*p* < 0.05 and ^∗∗^*p* < 0.01 refer to the liver and ^#^*p* < 0.05 and ^##^*p* < 0.01 refer to the kidneys, using one-way ANOVA followed by the Student-Newman-Keuls test.

In Figure 2, we show that mice of the control group that received a normal diet exhibited lower TEAC in the liver than that in the kidneys.

In CCl_4_-treated mice, there was a significant reduction of TEAC in the kidneys. This effect was expected; it has previously been shown that CCl_4_ increases the quantity of reactive species [30, 31].

In the group that received vitamin E, the TEAC was statistically higher (*p* < 0.05) in the kidneys and liver than that in the kidneys and liver of the control group. There was no statistically significant difference (*p* > 0.05) between animals treated with vitamin E and vitamin E + CCl_4_. Thus, vitamin E reduced the oxidative stress associated with CCl_4_. This was also in the animals treated with GB. The antioxidant activity of GB has been previously demonstrated *in vitro* [9, 12]. Therefore, we assume that this activity also occurs *in vivo*; consequently, the activity of GB was similar to that of vitamin E.

No other *in vivo* studies have used this assay to determine the antioxidant capacity of seaweed; *in vitro* studies have been performed, such as that carried out by Agregán and colleagues [32]. Those authors evaluated the *in vitro* antioxidant activity of extracts from *Ascophyllum nodosum*, *Bifurcaria bifurcata*, and *Fucus vesiculosus* and observed that *F. vesiculosus* was better able to inhibit radical formation compared with the other algae. However, in that study, the characteristic or component of the seaweeds responsible for the antioxidant capacity was not determined. Sulfated polysaccharides and phenolic compounds have been shown to be the main antioxidant agents in *G. birdiae* [9, 12].

### 3.3. Antioxidant Activity of GB *In Vivo*

In addition to the antioxidant compounds, GB may also act in the defense mechanisms inherent to the organism, for example, increasing the activity of antioxidant enzymes. Regarding CCl_4_-induced toxicity, the balance between the production of reactive species and antioxidant enzymes may be lost, generating oxidative stress. Therefore, biomarkers of oxidative stress (SOD, CAT, and GR) were evaluated in liver and kidney homogenates from untreated mice and mice treated with the different compounds (CCl_4_, *G. birdiae*, and vitamin E).

**Figure 3:**
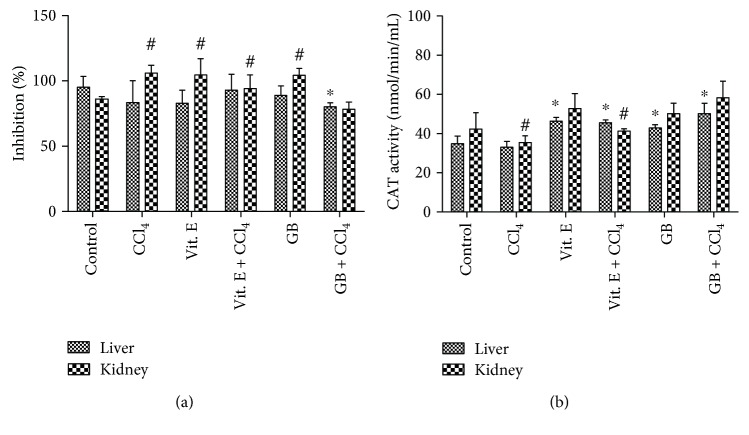
(a) Percentage inhibition of the antioxidant activity of superoxide dismutase (SOD). ∗ and # indicate a significant difference between groups versus control. ^∗^*p* < 0.05 refers to the liver and ^#^*p* < 0.05 refers to the kidneys, using one-way ANOVA followed by the Student-Newman-Keuls test. (b) Catalase activity. ∗ and # indicate a significant difference between groups versus control. ^∗^*p* < 0.05 refers to the liver and ^#^*p* < 0.05 refers to the kidneys, using one-way ANOVA followed by the Student-Newman-Keuls test.

Figure 3(a) shows that no statistically significant increase in SOD activity was observed in the liver of animals from the negative control group (without treatment) (*p* > 0.05) compared to those from the other groups, with the exception of GB + CCl_4_-treated animals, in which, a decrease in SOD activity was observed. Thus, the groups treated with CCl_4_, vitamin E, CCl_4_ + vitamin E, and GB demonstrated no change in liver SOD activity. All treatments significantly increased (*p* < 0.05) the SOD activity in the kidney in a similar way, that is, there was no difference between treatments. An exception was observed in the group treated with GB + CCl_4_, in which the SOD activity was similar to that observed in the control group.

SOD activity has not been reported in other studies evaluating the action of the red seaweed ex vivo. Nevertheless, Wu and colleagues [33] showed that animals treated with sulfated polysaccharides from the brown algae *Hizikia fusiformis* had increased SOD activity when compared with those treated with CCl_4_.

Different studies have evaluated the activity of this enzyme in vegetable sources. For example, Melo-Silveira and colleagues [34] showed that animals fed with corncob antioxidant polysaccharide-rich extract, even when exposed to CCl_4_, had similar SOD activity to those observed in mice treated with CCl_4_ + vitamin E. The authors concluded that the extract possessed vitamin E-like activity in terms of SOD activity, possibly due to the presence of antioxidant compounds. Liu and colleagues [35] also showed that polysaccharides from *Arctium lappa* L. were able to decrease the SOD activity in mice when exposed to CCl_4_.

It is important to note that different nutrients, such as natural antioxidants and other substances, do not necessarily affect all the enzymes in the redox system in the same way and to the same degree. Antioxidant enzymes may respond independently to different radical inducers and therefore may respond in different ways [36].

Figure 3(b) shows the activity of the liver enzyme catalase; there was no statistically significant difference (*p* > 0.05) between the control group and the group treated with CCl_4_.

In the kidney (Figure 3(b)), the presence of CCl_4_ significantly decreased CAT activity. Furthermore, vitamin E was not able to reverse this effect. Conversely, GB significantly increased CAT activity in the absence and presence of CCl_4_. These data show that the antioxidant agents present in GB were much more effective than vitamin E. However, in other studies, not all extracts evaluated were more effective than vitamin E. For example, Rajesh and colleagues [37] showed that extracts from *Mentha arvensis* were incapable of reversing the effect of CCl_4_ in terms of CAT activity. We believe that GB, due to the polysaccharides and phenolic compounds that act as antioxidants [12], has a broader antioxidant action than vitamin E, making it more effective. Vitamin E converts free radicals into more stable species through the donation of a hydrogen atom [38].

In another study, the effect of the algal extract on CAT and SOD activities was evaluated. Extracts of the green seaweed *Ulva lactuca* were found to decrease the activity of those enzymes. However, it is worth noting that this was evaluated using hypercholesterolemic animals, which may increase enzymatic activities due to oxidative stress.

**Figure 4:**
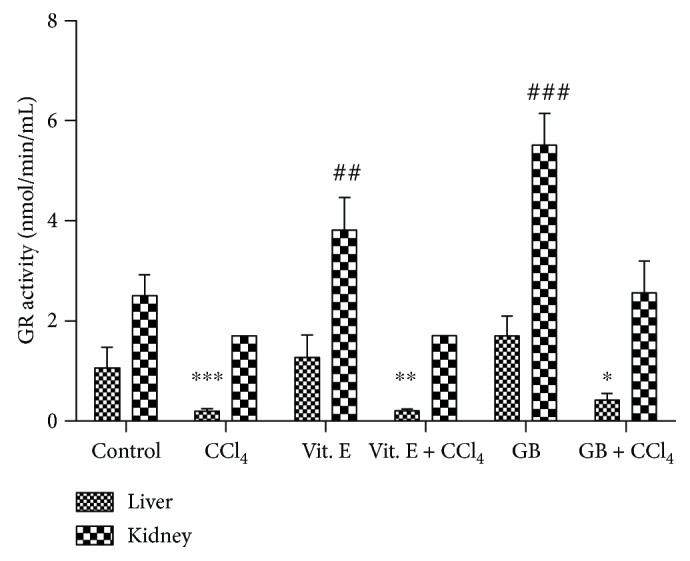
Glutathione reductase activity. ∗ and # indicate a significant difference between groups versus control. ^∗^*p* < 0.05, ^∗∗^*p* < 0.01, and ^∗∗∗^*p* < 0.001 refer to the liver and ^##^*p* < 0.01 and ^###^*p* < 0.001 refer to the kidneys, using one-way ANOVA followed by the Student-Newman-Keuls test.

Figure 4 shows the GR activity in the different groups. Antioxidant agents (vitamin E or GB) promoted a significant increase in GR activity in the kidneys. Notably, GB increased the GR activity almost three-times higher than that observed in the negative control group. Only GB increased GR activity in the liver; however, this was of lower intensity than that observed in the kidneys.

Conversely, the presence of CCl_4_ significantly decreased GR activity, especially in the liver. In this case, even the presence of antioxidants was not capable of restoring the GR activity. Regarding the kidneys, a similar pattern was observed. However, in this case, GB significantly protect the GR activity, whereas Vit. E was not effective.

Although the effect of seaweed on GR activity has not been reported in previous *in vivo* studies, we identified studies that evaluated the effect of plant extracts. In those studies, plant extracts seem to behave in a similar way when conferring protection, even in the presence of compounds that induce oxidation, such as CCl_4_. For example, Marineli and colleagues [36] and Ting and colleagues [39] observed a decrease in GR activity caused by an oxidative inducer and an increase caused by the administration of extracts from *Salvia hispanica* L. and with the seed oil of *Hippophae rhamnoides* L., respectively.

Therefore, the results of those studies are consistent with the findings of the present research. Thus, in the presence of GB, there was a significant increase in GR activity, which was reduced following the administration of CCl_4_, since GR would combat the deleterious effects of this oxidative inducer. Thus, it is suggested that GB can block the deleterious effects of reactive species resulting from CCl_4_ biotransformation.

### 3.4. Histological Analysis

Histopathological studies were performed in mice to assess the effect of CCl_4_, GB, and vitamin E administration on liver and kidney tissues and to verify whether tissue damage is reduced following administration of the toxic compound CCl_4_ in association with the seaweed and vitamin E.

**Figure 5:**
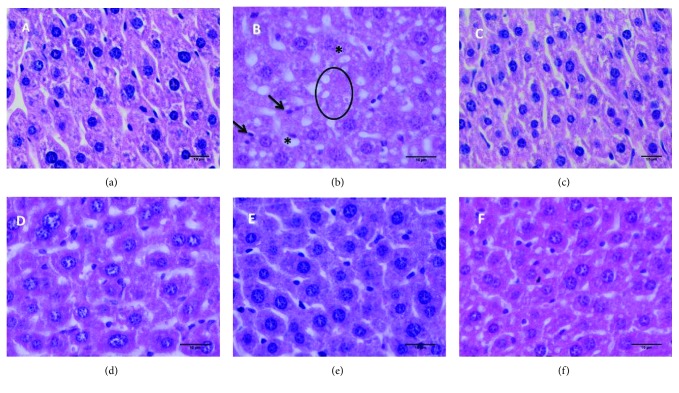
Histopathological changes in the mouse liver (hematoxylin and eosin stain). (a) Control mouse liver. (b) Mouse liver pretreated with CCl_4_. (c) Mouse liver pretreated with GB. (d) Mouse liver pretreated with GB + CCl_4_. (e) Mouse liver pretreated with vitamin E. (f) Mouse liver pretreated with vitamin E + CCl, magnification 20x. Bar: 10 *μ*m. Circle: necrosis; arrow: pycnosis; asterisk: cell vacuoles.

Figure 5 shows histological sections of livers. In the negative control group (Figure 5(a)), normal hepatocytes with preserved cytoplasm and nucleus can be observed. The same characteristics were observed in the liver of the animals treated with GB (Figure 5(c)) and vitamin E (Figure 5(e)). This indicates that GB is not toxic to the animals. In contrast, the liver sections of mice that received CCl_4_ (Figure 5(b)) contain pyknotic nuclei, vacuolized cells, liver damage with moderate to severe hepatocellular degeneration, and necrosis. When CCl_4_ and GB (Figure 5(d)) or vitamin E (Figure 5(f)) were administered, these parameters were all decreases, indicating hepatic injury. These results support the data obtained from the antioxidant assays in the present study and reinforce that the compounds present in the seaweed were able to minimize the deleterious effects of CCl_4_.

As in the present study, Wu and colleagues [33] evaluated the protective effect of sulfated polysaccharides from the brown alga *Hizikia fusiformis* in the liver of mice and also obtained positive results.

**Figure 6:**
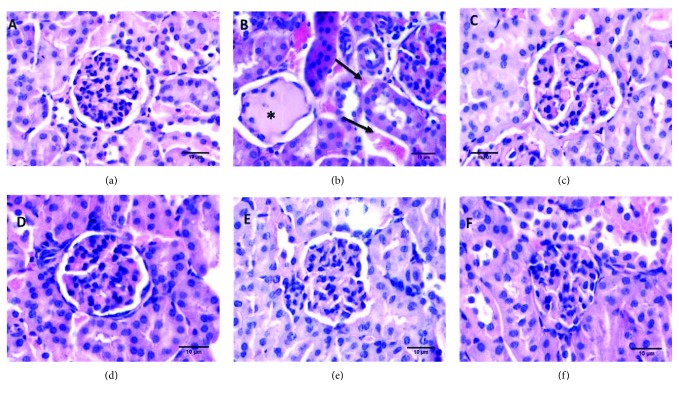
Histopathological changes in the mouse kidney (hematoxylin and eosin stain). (a) Control mouse kidney. (b) Mouse kidney pretreated with CCl_4_. (c) Mouse kidney pretreated with GB. (d) Mouse kidney pretreated with GB + CCl_4_. (e) Mouse kidney pretreated with vitamin E. (f) Mouse kidney pretreated with vitamin E + CCl_4_, magnification 40x. Bar: 10 *μ*m. Asterisk: necrosis; arrow: hematosis (intense vascularization).

Figure 6 shows histological sections of the kidneys. The kidneys of animals in the control group (Figure 6(a)) and those treated with GB (Figure 6(c)) and vitamin E (Figure 6(e)) presented well-preserved glomerulus. In contrast, kidney sections from mice treated with CCl_4_ (Figure 6(b)) contained renal tubules characterized by necrosis and loss of the glomerular borders, which are suggestive of inflammation and intense vascularization. However, the histopathological lesions observed following CCl_4_ administration were minimized with the administration of GB (Figure 6(d)) and vitamin E (Figure 6(f)). Rodrigues and colleagues [40] also observed that extracts from the red alga *Hypnea musciformis* also protected the renal tissue from CCl_4_-induced damage. The authors stated that the protective action of the alga is mainly due to the presence of the antioxidant sulfated polysaccharides in the extracts. Therefore, we believe that GB protected the renal tissues of the animals via the potential antioxidants synthesized by it, including sulfated polysaccharides.

### 3.5. Cytotoxicity and Inhibitory Effect of GB Extracts on Adipogenesis in 3T3-L1 Cells

Interestingly, animals that received GB presented a lower weight gain compared with those of the control group (Figure 1). An ethanolic extract of the red alga *Gelidium amansii* inhibited lipid accumulation in 3T3-L1 adipocytes [41] and prevented mice from gaining weight in diet-induced obesity [26]. In addition, Kang and coworkers [42] evaluated ethanolic extracts obtained from 27 different algal species as potential antiobesity agents by testing their effect on the adipogenic differentiation of 3T3-L1 cells and on animal weight gain. Twelve extracts decreased the rate of differentiation of 3T3-L1 cells into adipocytes, and the three most potent extracts were obtained from three red seaweeds. The most potent extract, obtained from the red seaweed *Plocamium telfairiae*, was also evaluated *in vivo*, and the data showed that this extract decreased the weight gain of rats fed with a hypercaloric diet. Other studies have shown that sulfated polysaccharides, obtained from aqueous algal extracts, exert antiobesity activity, because they decreased the rate of differentiation of 3T3-L1 cells into adipocytes [43, 44].

These data show that antiobesity molecules can be obtained from seaweed by both ethanolic and aqueous extractions. Therefore, to verify where the main molecules responsible for the effect of GB on weight gain were derived, we obtained two extracts of this alga, ethanolic extract and aqueous extract.

**Figure 7:**
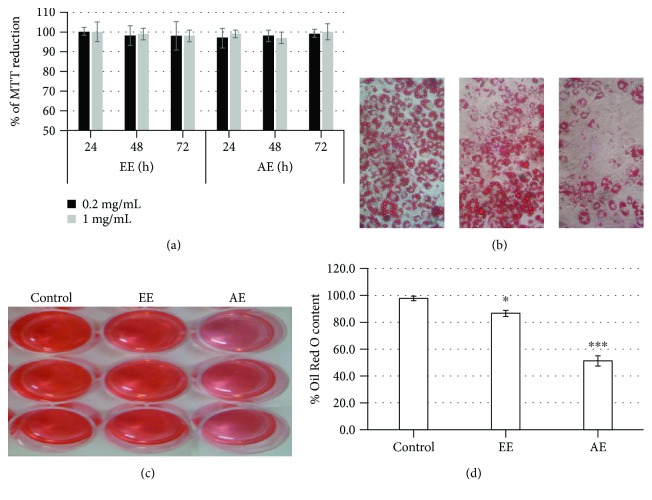
Effect of GB extracts on 3T3-L1 cells. (a) Cytotoxicity of GB extracts. Cytotoxicity of extracts was determined using the MTT assay. The cells were seeded at a density of 1 × 10^5^ cells/mL in 96-well plates and treated with GB extracts for 24, 48, and 72 h. The optical density of cells not exposed to extracts was considered as 100% of relative MTT reduction. (b) GB extracts inhibit intracellular lipid accumulation in 3T3-L1 adipocytes. 3T3-L1 cells stained with Oil Red O after 15 days of treatment with the extracts. Several drops of lipids can be observed within the cytoplasm of cells that differentiated into adipocytes. Bar = 20 *μ*m. (c) Overview of wells following the elution of Oil Red O from within the cells. Note that the cells treated with AE are stained with a lighter color. (d) Stained oil droplets were dissolved with isopropanol and evaluated by spectrophotometric analysis at 510 nm. The optical density of cells treated only with MDI was considered to be 100% relative lipid content. Values are expressed as mean ± standard deviation. ^∗^*p* < 0.05; ^∗∗∗^*p* < 0.001.

To investigate the cytotoxic effect of GB extracts, 3T3-L1 cells were treated with different concentrations (0.2 and 1.0 mg/mL) of these extracts and cellular viability was assessed via MTT assay. As shown in Figure 7(a), treatment with EE and AE for 24, 48, or 72 hours did not affect the capacity of 3T3-L1 to reduce MTT.

The effect of AE and EE (0.2 mg/mL) on adipogenesis was assessed using 3T3-L1 cells in the presence of adipogenic medium (MDI: dexamethasone, IBMX, insulin, and fetal bovine serum). The size and number of lipid droplets in 3T3-L1 adipocytes after AE and EE treatment were visualized using microscopy. After approximately 3 days of incubation in the presence of MDI, 3T3-L1 cells started to exhibit adipocyte morphology, including intracellular accumulation of fat droplets (data not shown). After 15 days, the cells were stained with Oil Red O and the number of droplets was higher in mature 3T3-L1 adipocytes (positive control). There was a slight decrease in the number and size of lipid droplets in mature 3T3-L1 adipocytes treated with EE, whereas a noticeable decrease was observed in mature 3T3-L1 adipocytes (Figure 7(b)).

Lipid accumulation in 3T3-L1 cells following AE treatment was quantified using Oil Red O staining. As shown in Figures 7(c) and 7(d), the amount of intracellular Oil Red O extracted from AE-treated 3T3-L1 cells corresponded to only 50% of the amount found in mature adipocytes (control, defined as 100% fat droplet content). Moreover, in the presence of EE, the level of Oil Red O decreased to only 10%.

Agregán et al. [32] evaluated ethanol extracts of 27 seaweeds, and not all extracts exhibited an antiadipogenic effect. This indicates that this is not an inherent activity of seaweed. To the best of our knowledge, no other study has evaluated the antiadipogenic effect of ethanolic extracts from seaweed of the genus *Gracilaria*. Therefore, a comparison of our data with those of other authors was not possible. Furthermore, although our data indicate a nonantiadipogenic action of EE, the EE was obtained from seaweed processed according to the methods used by fishermen, as we aimed to evaluate the seaweed as it is consumed. During processing of seaweed by the fishermen, the samples were exposed to the sun until they became discolored. This process may have destroyed compounds responsible for the antiadipogenic activity of EE. In future studies, we hope to verify this hypothesis by evaluating the EE obtained from GB that has not been cleared/dried by the sun.

### 3.6. Chemical Composition of Methanolic and Aqueous Extracts of GB

As the GB extracts presented different levels of antiadipogenic activity, we characterized and compared the main components of these extracts.

Table 2 shows that the EE of GB is composed mainly of phenolic compounds. A low content of sugar was also identified, corresponding to galactose. Gomes and coworkers [21] evaluated the sugar and phenolic compound content of alcoholic extracts from two red algae (*Botryocladia occidentalis* and *Acanthophora spicifera*) and detected only phenolic compounds in these extracts. This corroborates our results. Ethanol, methanol, and propanone are generally used to precipitate polysaccharides and oligosaccharides; therefore, we believe that GB polysaccharides were not fully solubilized in the 80% ethanol solution.

Conversely, the AE consists mainly of sugar and sulfate (Table 2), which is indicative of the presence of sulfated polysaccharide in the extract. Data on the monosaccharide composition from the present study are very similar to those reported by Fidelis and coworkers [12], which further indicates that AE is rich in sulfated polysaccharides.

**Figure 8:**
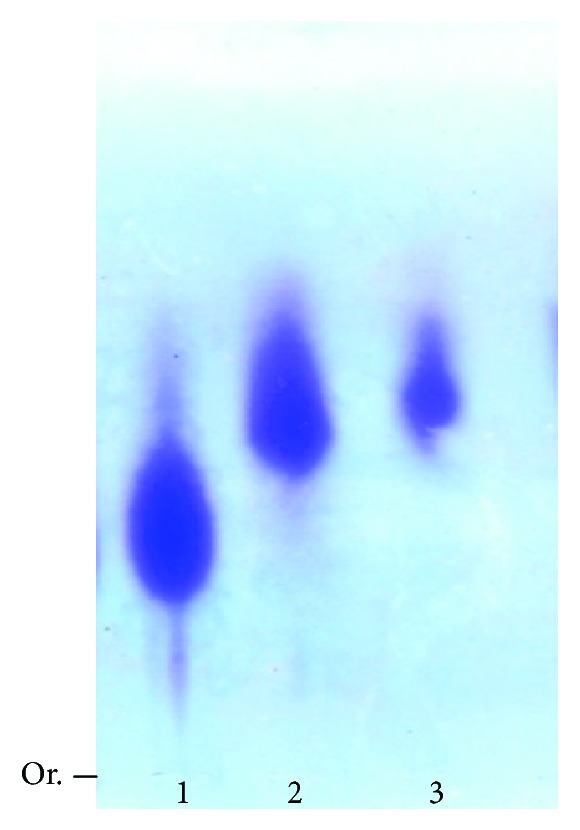
Electrophoresis in 0.05 M PDA buffer, pH 9.0, of sulfated polysaccharides obtained from seaweeds. 1: heterofucan (50 *μ*g) from brown seaweed *Spatoglossum schroederi*; 2: AE (500 *μ*g); and 3: AE (250 *μ*g). Or.: origin.

To confirm whether the sulfate was covalently linked to the polysaccharide in AE, this extract was subjected to electrophoresis in agarose gel and the gel was stained with toluidine blue.  Figure 8 shows that the sulfated polysaccharide (fucan from brown seaweed *Spatoglossum schroederi*), which was used as a standard, exhibited a purple coloration, typical of sulfated polysaccharides when stained with toluidine blue [45]. The AE also generated an electrophoretically mobile purple-colored band. This indicates that AE contains sulfated polysaccharide. In addition, even when electrophoresis was carried out at a higher concentration, only one electrophoretic band was visible. This indicates that a single type of sulfated polysaccharide predominates, which corroborates the data of [46], who obtained an aqueous extract of *G. birdiae* and showed that this extract contained only one type of sulfated polysaccharide.

## 4. Conclusion

The results of the present study indicate that the seaweed *G. birdiae* protected both the liver and the kidneys of mice from damage caused by CCl_4_, indicating that the seaweed studied exhibits a protective action *in vivo*, possibly due to its antioxidant capacity. In addition, GB decreased the rate of animal weight gain and its aqueous extract showed antiadipogenic activity; these effect may be due to the action of the sulfated polysaccharides synthesized by this seaweed.

## Figures and Tables

**Table 1:** Glycemia dosage of the mice.

Treatment	Glucose (mg/dL)
Control	303.50 ± 19.69^a^
CCl_4_	303.00 ± 38.50^a^
Vit. E	237.25 ± 21.28^b^
Vit. E + CCl_4_	286.38 ± 42.43^a^
GB	242.83 ± 21.86^b^
GB + CCl_4_	295.17 ± 20.37^a^

^a,b^Different letters indicate a significant difference between each treatment by two-way ANOVA followed by Bonferroni posttest (*p* < 0.05).

**Table 2:** Sulfate, protein, and phenolic compounds and molar ratio of monosaccharide constituents of GB extracts.

Sample	Sugar (%)	Sulfate (%)	Protein (%)	Phenolic compound (%)	Monosaccharide molar ratio				
					Gal	Glu	Ara	Xyl	Gluc A
AE	88.2 ± 1.2	11.1 ± 0.6	0.1 ± 0.05	0.0 ± 0.02	1.0	0.2	0.6	0.5	0.9
EE	2.0%	nd	nd	98%	1	nd	nd	nd	nd

Gal: galactose; Glu: glucose; Xil: xylose; Ara: arabinose; Glu A: glucuronic acid; nd: not detected.

## Data Availability

The data used to support the findings of this study are available from the corresponding author upon request.
